# Beyond the MSC Paradigm: Toward Unified Concepts, Methods and Applications in Secretome Research

**DOI:** 10.3390/biology15211924

**Published:** 2026-10-23

**Authors:** Ernesto Romero-López, Pamela Pastrana-Peralta, Jose Correa-Basurto, Adriana Romo-Pérez, Aurora González-Fierro, Alfonso Dueñas-González

**Affiliations:** 1 Division of Research, Immunotherapy Regenerative Medicine, Puerto Vallarta 48335, Mexico; dr.ernestoromero@immunotherapymx.com; 2 Division of Basic Research, Instituto Nacional de Cancerología, Mexico City 14080, Mexico; pam_pastrana@ciencias.unam.mx (P.P.-P.); aufierro@hotmail.com (A.G.-F.); 3 Laboratorio de Diseño y Desarrollo de Nuevos Fármacos e innovación Biotecnológica, SEPI-ESM, Instituto Politécnico Nacional, Mexico City 11340, Mexico; corrjose@gmail.com; 4 Instituto de Química, Universidad Nacional Autónoma de México, Mexico City 04510, Mexico; adriana.romo@iquimica.unam.mx; 5 Instituto Materno Infantil del Estado de México, Toluca 50170, Mexico

**Keywords:** secretome, nanostructured secretome, extracellular vesicles, unconventional protein secretion, mesenchymal stromal/stem cells, multi-omics, liquid biopsy, cell-free therapeutics, cross-kingdom biology, artificial intelligence

## Abstract

**Simple Summary:**

Cells send messages to each other by releasing a mix of proteins, tiny bubbles carrying cargo, fats, and other small molecules. Scientists call this mix the secretome. So far, most research has focused on just one type of human stem cell, so we do not fully understand how this process works in other human cells, plants, fungi, or bacteria. This review brings together the main ideas, lab methods, and real-world uses of the secretome across all these living things. It shows that while the basic ways of releasing these mixtures are similar, each organism has its own methods. New tools that track newly made proteins, study single cells, and use computers are helping us map these messages in more detail. These mixtures are already used in industrial enzymes and crop protection and are actively being evaluated in preclinical models and clinical trials for early disease detection, cell-free healing, and cancer therapies. Studying more than one stem cell type will produce better maps of cell communication and more reliable products. This wider approach will help society by allowing earlier disease detection, safer healing treatments, and sustainable progress in medicine, industry and farming.

**Abstract:**

The secretome has evolved from a protein-centered concept into a dynamic and multidimensional communication system comprising soluble proteins, extracellular vesicles, metabolites, lipids, nucleic acids, and other bioactive components. Despite major advances in secretome research, the field remains fragmented across biological systems and has been particularly dominated by studies of mesenchymal stromal/stem cells (MSCs). This review provides a unifying perspective on secretome biology across mammalian, plant, fungal, and bacterial systems. We examine the evolution of secretome definitions, classical and unconventional secretion pathways, and emerging analytical approaches, including mass spectrometry, metabolic labeling, single-cell and spatial secretomics, multi-omics integration, and artificial intelligence. We further discuss applications in biomarker discovery, liquid biopsy, regenerative medicine, cancer biology, biotechnology, and cell-free therapeutics, together with current challenges in standardization, reproducibility, manufacturing, and clinical translation. Moving beyond an MSC-centered framework toward tissue-, cell-type-, and organism-resolved secretome mapping may enable more comprehensive communication atlases and support the development of predictive biomarkers, precision therapeutics, and rationally engineered secretome-based products.

## 1. Introduction

The concept of the secretome has evolved from a specialized term in bacterial proteomics to a central framework for understanding intercellular communication across biological systems. Tjalsma and colleagues first introduced the term in their genomic analysis of Bacillus subtilis, where it referred to secreted proteins and the cellular machinery that exports them [1,2]. Later definitions broadened to include extracellular vesicles (EVs), lipids, nucleic acids, and other nanostructured components, highlighting that cellular communication occurs through a complex, multicomponent nanostructured secretome [3,4,5]. Over the past two decades, secretome research has expanded substantially, driven by advances in mass spectrometry-based proteomics, metabolic labeling, and growing interest in cell-free therapeutics. Despite this growth, the recent literature has mainly focused on the secretome of mesenchymal stromal/stem cells (MSCs). Studies have examined MSC-derived conditioned media and EV preparations for their regenerative, immunomodulatory, anti-inflammatory, and pro-angiogenic properties across diverse pathologies [5,6,7]. Additional research has explored MSC secretomes in cancer, showing both anti- and pro-tumorigenic effects that vary with tissue source, priming conditions, and EV content [8,9]. Although this MSC-focused literature has provided valuable preclinical insights, it has also created an unbalanced representation of the broader secretome field.

The predominance of the MSC paradigm presents several limitations. First, MSC secretomes show considerable molecular and functional heterogeneity due to donor variability, tissue origin, culture conditions, passage number, and preconditioning strategies. This complicates reproducibility and potency assessment [5,7,10]. Second, excessive focus on MSCs may obscure important secretome biology in other mammalian cell types, as well as in plants, fungi, and bacteria, where secreted components are essential for defense, virulence, symbiosis, and environmental adaptation [11,12]. Third, methodologies developed in MSC and cancer research are not always directly applicable to other domains [13]. Furthermore, the emphasis on regenerative applications has left conceptual, comparative, and cross-kingdom perspectives underdeveloped.

This review seeks to advance beyond the MSC-focused paradigm by offering a comprehensive perspective on secretome biology. The objectives are to consolidate core concepts, including the expanding nanostructured view of the secretome; synthesize analytical and computational methods across biological domains, emphasizing shared principles and system-specific adaptations; and survey applications encompassing mammalian physiology and pathology, as well as microbial, fungal, plant, and translational systems.

### Literature Search Strategy

We searched PubMed, Web of Science, and Scopus for literature published between January 2000 and May 2026. The primary search terms were ‘secretome’, ‘nanostructured secretome’, ‘unconventional protein secretion’, ‘extracellular vesicles’, ‘apoplast secretomics’, and ‘bacterial secretion systems’. Each was paired with qualifiers such as ‘mesenchymal stem cell’, ‘cancer’, ‘fungi’, ‘plant’, ‘mass spectrometry’, and ‘GMP manufacturing’. Bibliometric analysis shows an exponential increase in global secretome research over the past two decades. Annual publications containing ‘secretome’ rose from fewer than 50 in 2006 to over 2000 by 2025. From 2012 to 2022, research focused mainly on MSC regenerative applications. The most recent four years (2022–2026) show a marked shift toward multi-omic, single-cell workflows, non-MSC lineages, and cross-kingdom comparative biology.

## 2. Evolving Concepts and Definitions of the Secretome

### 2.1. Historical Trajectory: From Cellular Machinery to Extracellular Products

Tjalsma and colleagues introduced the term “secretome” in 2000 regarding the Gram-positive bacterium Bacillus subtilis [1]. In their genome-based survey, they defined the secretome as the set of proteins destined for export and the cellular machinery that secretes them. This initial definition focused strictly on proteins and secretion machinery, excluding extracellular vesicles and non-membranous nanoparticles, which the field later incorporated as it evolved into its current multi-component framework.

A decade later, Agrawal and colleagues refined the concept, especially in plant biology, by proposing a more product-oriented definition: “the global group of secreted proteins into the extracellular space by a cell, tissue, organ, or organism at any given time and conditions through known and unknown secretory mechanisms involving constitutive and regulated secretory organelles” [2]. Traditional definitions focused almost exclusively on soluble proteins. However, evidence shows that cells also release a diverse range of nanoparticles, leading to the concept of the “nanostructured secretome.” This broader view acknowledges that cellular communication occurs not only through free proteins but also via a complex mixture of molecular and particulate entities [3,4,5,14].

### 2.2. Pathways of Export: Classical vs. Unconventional Secretion

Secreted proteins reach the extracellular space via distinct pathways determined by cellular organization. In eukaryotic cells, including mammalian, plant, and fungal species, classical secretion depends on an N-terminal signal peptide that targets nascent polypeptides to the endoplasmic reticulum (ER). COPII-coated vesicles then transport these proteins to the Golgi apparatus for processing and sorting. The processed cargo is delivered to the plasma membrane and released through exocytosis [15,16,17]. Unconventional protein secretion (UPS) includes several mechanisms that bypass the ER–Golgi pathway. These include direct translocation across the plasma membrane, such as pore-mediated export of FGF2; ABC-transporter-mediated export; incorporation into endosomal or multivesicular body-derived vesicles or secretory autophagy intermediates; and release via plasma-membrane-derived microvesicles [18,19,20]. UPS is especially prominent for leaderless proteins that lack a classical signal peptide. In plants, leaderless secreted proteins may make up more than half of the experimentally detected secretome [2,19], highlighting the quantitative significance of unconventional secretion in some systems.

In contrast, bacterial cells lack an endoplasmic reticulum, Golgi apparatus, or endolysosomal network. In prokaryotes, classical secretion refers to the Sec (secretion) and Tat (twin-arginine translocation) pathways. These pathways use distinct cytoplasmic chaperones and membrane-embedded translocases to export proteins across the inner plasma membrane into the periplasm or extracellular environment, bypassing organelle-associated packaging [15,16].

### 2.3. The Emerging Conceptual Framework of the ‘Nanostructured Secretome’

Traditional definitions focused almost exclusively on soluble proteins. Growing evidence shows that cells also release a diverse repertoire of nanoparticles, defining the “nanostructured secretome” [3]. In this expanded view, the secretome comprises:

1. Soluble proteins, peptides, metabolites, and small molecules.

2. Extracellular vesicles (exosomes, micro vesicles, apoptotic bodies) carrying proteins, lipids, nucleic acids, and metabolites.

3. Non-membranous nanoparticles such as lipoproteins, exomeres, supermeres, and macromolecular complexes.

Extracellular vesicles are released through distinct biogenesis routes:

**Exosomes (30–150 nm):** These vesicles originate from the endosomal system. Early endosomes mature into multivesicular bodies (MVBs) through inward budding of the limiting membrane, generating intraluminal vesicles (ILVs). This process occurs via ESCRT-dependent machinery (ALIX, TSG101) or ESCRT-independent routes involving ceramides and tetraspanins. Mature MVBs fuse with the plasma membrane via Rab GTPases (e.g., Rab27) and SNARE proteins, releasing ILVs as exosomes.

**Micro vesicles/ectosomes (100–1000 nm):** These vesicles form by direct outward budding and fission of the plasma membrane driven by membrane lipid asymmetry, cytoskeletal remodeling (actin–myosin), calcium signaling, and small GTPases like ARF6 and RhoA [21,22,23].

Additional secreted nanoparticles include lipoproteins assembled in the endoplasmic reticulum and Golgi apparatus, as well as non-membranous particles such as exomeres and supermeres. Although their molecular profiles suggest specialized functions, the mechanisms behind their biogenesis remain poorly understood. The literature debates whether these particles are truly discrete biological entities or fragments of a continuous non-vesicular matrix. Consequently, their structural classification and distinction from classical extracellular vesicle (EV) pathways remain under investigation [3,24,25]. The secretome is considered the overarching extracellular output, encompassing functionally coherent subsets. These include established structural and functional networks such as the matrisome, adhesome, and receptorome. The term ‘nanostructured secretome’ offers a comprehensive framework for understanding multi-component extracellular signaling. However, it is a recent conceptual development and lacks widespread agreement on strict inclusion criteria. Debate continues over whether certain non-membranous nanoparticles, including exomeres and supermeres, are discrete, actively sorted biological entities or continuous non-vesicular material co-isolated during enrichment protocols.

**Figure 1:**
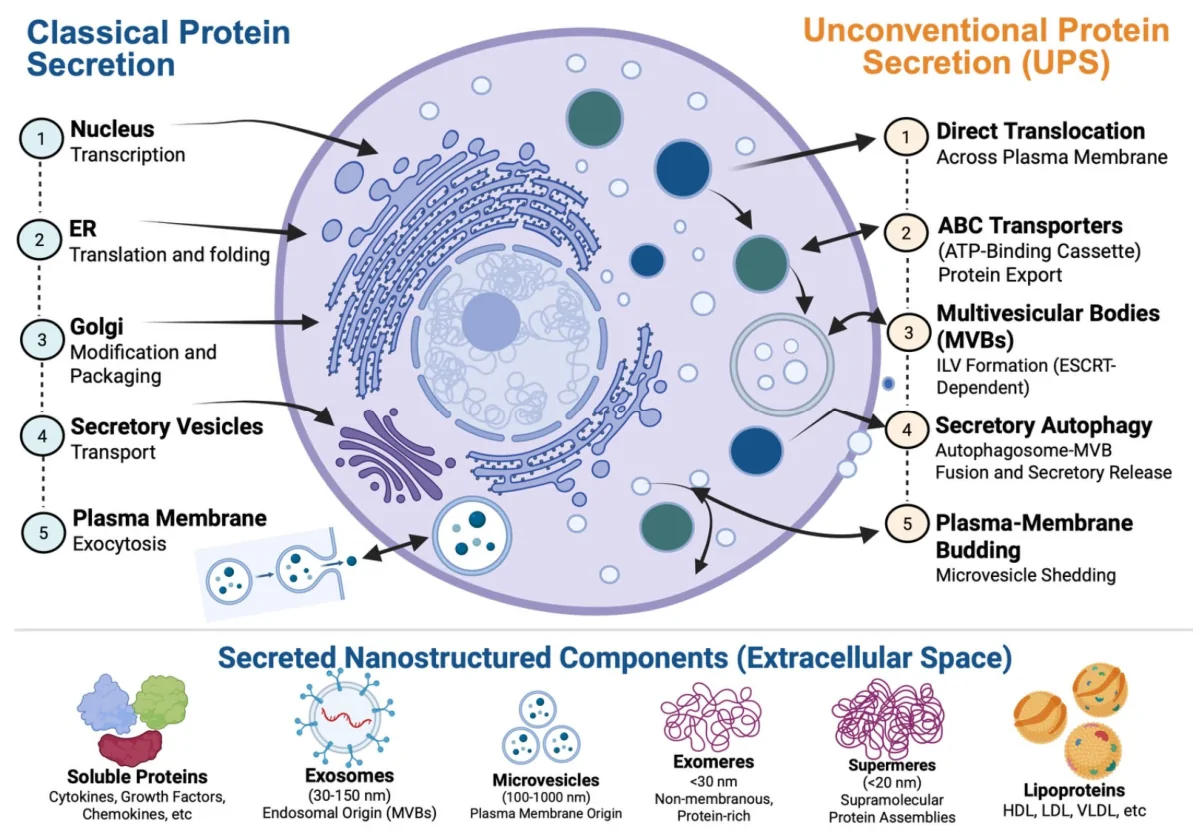
Classical and unconventional secretion pathways and components of the nanostructured secretome. Overview of classical and unconventional secretion pathways and nanostructured secretome components. Classical secretion relies on the nucleus, endoplasmic reticulum (ER), Golgi apparatus, and vesicular transport for terminal exocytosis. Unconventional protein secretion (UPS) bypasses the Golgi apparatus through mechanisms such as direct translocation across the plasma membrane, ATP-binding cassette (ABC) transporters, multivesicular bodies (MVBs) with intraluminal vesicle (ILV) formation, secretory autophagy via autophagosome-MVB fusion, and plasma membrane budding for microvesicle release. The lower panel depicts secreted nanostructured components in the extracellular space, including soluble proteins (cytokines, growth factors, chemokines), exosomes (30–150 nm), microvesicles (100–1000 nm), exomeres (<30 nm), supermeres (<20 nm), and lipoproteins (HDL, LDL, VLDL).

Figure 1 shows a representation of the “nanostructured” secretome and export pathways.

Classical secretion depends on the nucleus, endoplasmic reticulum, Golgi apparatus, and vesicle axis for terminal exocytosis. In contrast, unconventional protein secretion bypasses the Golgi apparatus through mechanisms including direct translocation, ATP-binding cassette (ABC) transporters, multivesicular bodies, secretory autophagy, and plasma membrane budding.

### 2.4. Overlapping “Omes” and Spatial Scales of Cellular Output

In addition, several specialized “omes” overlap with or are nested within the secretome:

(i) Matrisome: the ensemble of extracellular matrix (ECM) and ECM-associated proteins (core structural components—collagens, proteoglycans, glycoproteins—plus regulators and affiliated proteins). Many matrisome components are secreted and therefore form a major subset of the secretome [26,27].

(ii) Adhesome: the network of proteins that mediate cell–cell and cell–matrix adhesion. A substantial fraction of adhesome components are surface-exposed or secreted and intersect with both the matrisome and the broader secretome [28,29].

(iii) Receptorome: the repertoire of cellular receptors. While primarily membrane-bound, many receptors are shed into the extracellular space (soluble receptors) and thus become part of the secretome [30,31].

(iv) Circulome: a more recent concept referring to circulating molecular and particulate components—including circulating DNA, EVs, and proteins—detectable in biofluids. In cancer, the malignant circulome represents the tumor-associated fraction of this systemic molecular output [32,33].

These related omics are not mutually exclusive. The secretome represents the overall extracellular output containing functionally coherent subsets. This output includes established structural and functional networks like the matrisome, adhesome, and receptorome. It also includes emerging concepts such as the circulome, which reflects systemic extracellular components but lacks the definitional clarity of established matrix-associated omes. The secretome can be viewed as the overarching extracellular “output” of a cell or tissue, while the matrisome, adhesome, receptome, and circulome highlight functionally coherent subsets or spatial scales of that output. Recognizing these intersections facilitates more precise experimental design and interpretation across biological systems. In summary, the concept of the secretome has evolved from a bacterial genomic prediction tool into a broad, multicomponent framework that encompasses soluble factors, vesicles, and other nanoparticles released by classical and unconventional routes. This conceptual maturation provides the foundation for the comparative and methodological discussions that follow.

## 3. Analytical and Computational Approaches: Toward Unified Methods

### 3.1. Sample Preparation

Secretome analysis faces common and system-specific technical challenges. In mammalian cell-culture models, including mesenchymal stem cell (MSC) and cancer systems, major obstacles include contamination from intracellular proteins released during cell death or lysis, a broad dynamic range from serum proteins or supplements, and dilution of secreted proteins in large volumes of conditioned medium. Addressing these issues requires stringent upstream handling, viability controls, and serum-free or serum-depleted conditions [13].

In plant systems, additional challenges arise from the rigid cell wall and the high proportion of leaderless secreted proteins. Cytoplasmic contamination often complicates apoplastic fluid isolation or cell-culture supernatant collection. Prediction tools trained on mammalian data often perform poorly on plant sequences. Microbial and fungal secretomes add complexities, including cell-wall-degrading enzyme activity, complex culture media, and the need to distinguish actively secreted effectors from passively released proteins [34,35]. Similar challenges occur in bacterial and fungal systems, where rapid growth and cell lysis hinder separation of authentic secreted proteins, such as virulence effectors and outer-membrane vesicle cargo, from intracellular contaminants [13,35,36,37].

Effective cross-system solutions require rigorous validation with contamination markers, such as lactate dehydrogenase or organelle-specific proteins, and extraction protocols tailored to the specific matrix composition. Standardizing collection timing, culture conditions, and post-collection processing across biological domains is essential to ensure reproducibility [13].

### 3.2. Separation and Enrichment Strategies

To address the low abundance of extracellular products and remove interfering medium components, raw preparations require dedicated downstream separation and concentration steps. Common workflows use efficient protein recovery methods, including trichloroacetic acid (TCA) or acetone precipitation, ultrafiltration, and advanced solid-phase sample preparation frameworks such as SP3-based protocols.

These methods isolate extracellular fractions from large volumes of conditioned media. Mechanical boundary segregation techniques, including sequential differential ultracentrifugation and size-exclusion chromatography, are increasingly incorporated into separation pipelines to partition the secretome matrix into distinct physical subpopulations. This approach separates membranous extracellular vesicles from soluble extravesicular proteins and macromolecules [13].

### 3.3. Detection and Profiling Technologies

Mass spectrometry (MS) remains fundamental to unbiased secretome analysis. Shotgun liquid chromatography-tandem mass spectrometry (LC-MS/MS) of concentrated conditioned media or biofluids enables comprehensive profiling of both soluble and vesicle-associated proteins, although its depth is constrained by dynamic range and contamination. Data-independent acquisition (DIA) and advanced fractionation strategies enhance reproducibility and quantification [13]. Affinity proteomics platforms, including antibody arrays, aptamer-based assays, and targeted immunoaffinity enrichment, provide increased sensitivity for detecting low-abundance cytokines and growth factors. However, these methods are inherently biased toward known targets and are less effective for identifying unconventional or species-specific components [13,14,38].

Metabolic labeling approaches, particularly bioorthogonal non-canonical amino acid tagging (BONCAT) with azidohomoalanine (AHA) or related analogs, enable selective enrichment of newly synthesized proteins even in the presence of serum [39]. Combined with click chemistry and mass spectrometry, BONCAT improves specificity for newly synthesized secreted proteins and can be integrated with pulsed stable isotope labeling by amino acids in cell culture (SILAC) for quantitative analyses [13]. Proximity biotinylation strategies, such as TurboID, BioID, or APEX variants expressed in a cell-type-selective manner, facilitate in vivo or co-culture labeling of secreted proteins. Biotinylated proteins can be recovered directly from plasma or conditioned media, enabling cell-of-origin resolution of the secretome in complex multicellular systems [40,41]. Single-cell secretomics is an emerging frontier. Microfluidic chambers, droplet-based assays, and recent mass spectrometry-compatible workflows that combine metabolic labeling with capillary micro-reactors now enable profiling of hundreds of secreted proteins from individual cells, revealing previously inaccessible heterogeneity in secretion programs [42].

### 3.4. Data Standardization and Computational Analysis

Several specialized resources facilitate secretome annotation and prediction. PlantSecKB and MetazSecKB compile predicted and curated secretomes for plants and metazoans, respectively, integrating tools such as SignalP, Phobius, TargetP, and others [43,44]. The Human Protein Atlas includes a dedicated secretome section [45]. The recent Secreted Protein Database (SEPDB) aggregates experimental secreted-protein datasets from serum, exosomes, and conditioned media across human, mouse, and rat and incorporates updated signal-peptide predictors such as SignalP 6.0 [46,47]. Classical prediction tools, including SignalP for signal peptides and SecretomeP for leaderless secretion, remain widely used but have notable limitations, especially for SecretomeP when applied to plant sequences [35].

Recent advances in machine-learning models, multi-tool consensus pipelines, and protein language models (PLMs) have substantially improved predictive accuracy. For example, deep-learning frameworks such as *DeepSecE* integrate PLM features with transformer modules to accurately resolve multiclass secretion signals and substrate sorting across diverse systems. These computational developments are essential for progressing beyond organism- or cell-type-centric catalogs toward unified, predictive models of the secretome across biological domains [48].

Future integration of secretomics with transcriptomics, lipidomics, glycomics, and single-cell multi-omics, supported by artificial intelligence-driven analysis and deconvolution algorithms, is beginning to yield systems-level perspectives on secretory networks. Emerging deep-learning predictors and multi-omics foundation models, such as scGPT, may further enable integration of heterogeneous molecular datasets and inference of complex cell-to-cell communication patterns [49]. Collectively, these approaches establish a computational framework for transitioning from descriptive secretome catalogs to predictive models of intercellular and inter-organ communication.

### 3.5. Functional Verification and Potency Assays

The diversity of biological systems and experimental objectives necessitates multi-tiered evaluation workflows to distinguish genuine functional secretome components from correlative artifacts or those resulting from cell damage. Each discovery approach balances coverage depth, specificity, physiological relevance, and technical feasibility in distinct ways. The selection of verification methods is strongly influenced by the organism under investigation, the requirement to preserve native secretion patterns, and the ability to achieve cell-of-origin resolution.

Table 1 summarizes the principal strengths, limitations, and current best-use cases of the most widely employed strategies. No single workflow is universally optimal. Mammalian regenerative and cancer studies often use serum-free, mass spectrometry-based workflows or BONCAT-MS strategies. In contrast, plant and microbial systems require customized extraction procedures and more cautious interpretation of leaderless proteins [13,35]. Consequently, cross-domain method transfer necessitates careful validation through orthogonal targeted bioassays, such as endothelial tube formation kinetics, real-time cell proliferation assays, or macrophage polarization screens, to establish biological potency [5,13].

### 3.6. Comparative Evaluation of Workflows

The diversity of biological systems and experimental objectives has led to multiple complementary workflows for secretome analysis. Each approach offers a distinct balance of coverage depth, specificity, physiological relevance, and technical feasibility. Method selection depends on the organism or sample type, the need to preserve native secretion patterns, the presence of contaminating proteins, and the requirement for cell-of-origin or single-cell resolution. Table 1 summarizes the main strengths, limitations, and best-use cases for the most widely used strategies. No single workflow is universally optimal. Mammalian regenerative and cancer research often use serum-free mass spectrometry (MS)-based workflows or bioorthogonal non-canonical amino acid tagging mass spectrometry (BONCAT-MS) strategies. In contrast, plant and microbial systems require customized extraction procedures and more rigorous interpretation of leaderless proteins. Therefore, cross-domain method transfer requires thorough validation.

### 3.7. Databases, Prediction Tools, and Multi-Omics/AI Integration

A range of specialized resources support secretome annotation and prediction. PlantSecKB and MetazSecKB provide predicted and curated secretomes for plants and metazoans by integrating tools such as SignalP, Phobius, and TargetP [43,44]. The Human Protein Atlas includes a dedicated secretome section [45]. The Secreted Protein Database (SEPDB) aggregates experimental secreted-protein datasets from serum, exosomes, and conditioned media across humans, mice, and rats, and incorporates updated signal-peptide predictors like SignalP 6.0 [46,47]. Classical prediction tools, including SignalP for signal peptides and SecretomeP for leaderless secretion, remain widely used, although SecretomeP shows clear limitations when applied to plant sequences [35].

Recent advances in machine-learning models, multi-tool consensus pipelines, and protein language models (PLMs) have substantially improved secretome prediction accuracy. For example, deep-learning frameworks like *DeepSecE* integrate PLM features with transformer modules to resolve multiclass secretion signals and substrate sorting across diverse systems. These developments are critical for moving from organism- or cell-type-centric catalogs to unified, predictive models of the secretome across biological domains [48].

Integrating secretomics with transcriptomics, lipidomics, glycomics, and single-cell multi-omics, supported by AI-driven analysis and deconvolution algorithms, is starting to provide systems-level perspectives on secretory networks. Emerging deep-learning predictors and multi-omics foundation models like scGPT may further enable integration of heterogeneous molecular datasets and inference of complex cell–cell communication patterns [49]. Together, these approaches establish a computational framework that advances the field beyond descriptive analyses.

Secretome catalogs toward predictive models of intercellular and inter-organ communication.

## 4. Secretome Biology Across Systems

### 4.1. Mammalian Secretomes: Physiological and Pathological Contexts

Virtually every cell type continuously releases a complex mixture of soluble factors, EVs, and other nanostructured components that shape local tissue microenvironments and enable long-range communication [40,50]. In pathological settings, particularly cancer and chronic inflammation, these secretory programs are dramatically reprogrammed and often become key drivers of disease progression.

Cancer cells possess an aberrant secretome compared to their non-malignant counterparts. This “cancer cell secretome” influences nearly every stage of tumorigenesis, from local invasion and stromal reprogramming to pre-metastatic niche formation and therapy resistance. Tumor cells secrete matrix-remodeling enzymes, growth factors (e.g., VEGF, TGF-β, HGF), cytokines, and EVs that recruit and educate cancer-associated fibroblasts (CAFs), endothelial cells, and immune populations. In turn, CAFs and other stromal cells release secretomes that support tumor growth, angiogenesis, and immune evasion [51]. Extracellular vesicles, especially tumor-derived exosomes, transfer oncogenic proteins, metabolites, and nucleic acids that promote proliferative signaling, metabolic rewiring, epithelial–mesenchymal plasticity, and immunosuppressive niche formation. Hypoxia-conditioned tumor EVs extend these effects systemically, conditioning distant organs for metastatic colonization [52].

In experimental models, this paracrine subversion is compounded by immune and stromal cell populations recruited to the tumor microenvironment (TME), particularly tumor-associated macrophages (TAMs), myeloid-derived suppressor cells, and regulatory T cells, which contribute to the local secretome [53]. TAMs secrete chemokines (CCL2, CCL5, CCL22) and cytokines that recruit additional immunosuppressive cells and induce T-cell exhaustion. M1-like macrophages can produce CXCL9/CXCL10 that support effector T-cell responses. The bidirectional CAF–TAM axis, mediated by soluble factors and EVs, organizes the immunosuppressive stroma [54]. Beyond the TME, macrophage- and other myeloid-derived secretomes modulate inflammation, tissue repair, and fibrosis in many physiological and pathological contexts [55,56].

Outside of the stem-cell and oncology paradigms, many non-stem mammalian cell types orchestrate specialized, tissue-specific secretory programs to maintain systemic homeostasis. Endothelial cells, for example, release a diverse secretome composed of gaseous mediators, growth factors, cytokines, and baseline EVs. These components regulate vascular tone, orchestrate physiological angiogenesis, control localized inflammation, and drive metabolic crosstalk with adjacent adipose tissues and peripheral organs. Chronic endothelial dysfunction leads to an aberrant, pro-inflammatory secretome that promotes systemic fibrosis, atherosclerosis, and metabolic disease [57].

Similarly, healthy fibroblasts and myofibroblasts secrete extracellular-matrix components, matrix metalloproteinases, and signaling factors that drive tissue remodeling in wound healing, fibrosis, and cancer [58,59]. Adipocytes and other metabolic cells release adipokines and EVs that influence insulin sensitivity, inflammation, and energy homeostasis [60,61]. Immune cells (macrophages, T cells, dendritic cells, neutrophils) deploy dynamic secretomes—cytokines, chemokines, proteases, and EVs—that coordinate innate and adaptive responses, tissue repair, and inflammation resolution [62,63]. Moving beyond the MSC-centric view reveals a rich landscape of cell-type-specific, context-dependent secretory biology central to tissue homeostasis and disease.

### 4.2. Stem-Cell and Regenerative Secretomes (MSC and Non-MSC Sources)—Placed in Broader Perspective

Stem-cell secretomes, particularly those derived from MSCs, have occupied a central position in regenerative medicine research for more than a decade. The therapeutic benefits historically attributed to live MSC engraftment, survival, and direct differentiation are now widely recognized to be mediated primarily by paracrine factors. This paradigm shift has driven intense clinical interest in cell-free therapeutics based on conditioned media or purified EV fractions as potentially safer, more scalable, and non-immunogenic alternatives to live-cell transplantation [64].

MSC secretomes exhibit potent immunomodulatory, anti-inflammatory, pro-angiogenic, anti-apoptotic, and anti-fibrotic activities [65,66]. Their composition is highly plastic and can be modulated by tissue source (bone marrow, adipose tissue, umbilical cord, Wharton’s jelly), culture conditions, passage number, and preconditioning strategies such as hypoxia, inflammatory cytokines, 3D culture, or pharmacological priming. Inflammatory priming often enriches the secretome in chemokines and immunomodulatory factors, while hypoxia enhances angiogenic and cytoprotective cargo [67,68]. This plasticity is both a strength, enabling tailored bioactivity, and a challenge for standardization and clinical translation. The profound molecular and functional heterogeneity highlights a critical tension within the current paradigm. Although native MSC secretomes have therapeutic traits that make them leading translational candidates, their instability complicates standardized biomanufacturing. Therefore, this natural heterogeneity requires strict upstream parameters, bio-priming strategies, and fully chemically defined media as essential prerequisites for any MSC-derived formulation to address these contradictions and achieve clinical readiness.

Other stem and progenitor populations also release functionally significant secretomes. Induced pluripotent stem cell (iPSC)-derived MSCs or mesenchymal progenitors produce secretomes that largely recapitulate the inflammatory-licensing responses of tissue-derived MSCs while offering greater scalability and reduced donor variability [69]. Importantly, regenerative secretomes are not restricted to MSCs. Neural stem/progenitor cells, hematopoietic stem cells, and endothelial progenitor cells secrete factors and EVs that support neuroprotection, hematopoiesis, or vascular repair, respectively. Tissue-resident stem/progenitor cells such as satellite cells, hair-follicle stem cells, or cardiac progenitors release localized secretomes that orchestrate niche maintenance and tissue-specific regeneration [70,71,72,73]. A unified view recognizes MSC and non-MSC stem-cell secretomes as powerful, tunable therapeutic tools while situating them within the larger continuum of mammalian secretory biology [74]. This perspective encourages comparative studies across cell types, more rigorous benchmarking of potency and reproducibility, and the rational design of next-generation secretome-based or secretome-targeted interventions that draw on the full diversity of cellular sources.

### 4.3. Microbial and Fungal Secretomes: Virulence, Symbiosis, Industrial Enzymes

Bacterial pathogens rely on dedicated protein secretion systems (Types I–IX in Gram-negative bacteria and specialized systems in Gram-positive species) to export virulence factors. These include toxins, adhesins, effectors that manipulate host cell signaling, and enzymes that degrade host tissues or scavenge nutrients. Because bacterial cells lack an endosomal system or Golgi apparatus to organize and package cargo, they rely on fixed, multi-protein macromolecular complexes spanning the cell envelope as directional conduits. Rather than mimicking transient eukaryotic vesicle sorting, bacterial Type I through IX secretion systems function as static nanomachines or molecular syringes. These systems are structurally classified by their configuration:

**One-step systems (Types I, III, IV, and VI)** form continuous trans-envelope channels that bypass the periplasm. For example, Type III (T3SS) and Type VI (T6SS) systems act as physical syringes, using a needle-like structure to inject effector proteins (such as P. aeruginosa ExoS) directly from the bacterial cytoplasm into host cells or competing microbes.

**Two-step systems (Types II, V, and IX) first depend on** inner-membrane Sec or Tat translocases for periplasmic delivery. Once in the periplasm, the Type II system (T2SS) uses a pseudopilus structure to push folded proteins through outer-membrane secretin pores, serving as the terminal outer-membrane checkpoint for soluble toxins.

**Specialized Outer-Membrane Budding complements** fixed channels by shedding outer-membrane vesicles (OMVs) in Gram-negative bacteria. These package periplasmic proteins, lipids, and nucleic acids to serve as a mobile transport mechanism similar to, but distinct from, eukaryotic extracellular vesicles. Bacterial pathogens rely on sophisticated macromolecular complexes spanning cell envelopes—specifically Types I through IX secretion systems in Gram-negative bacteria, and Sec/TAT pathways or Type VII systems in Gram-positive species—to translocate virulence factors into host cells or the extracellular matrix. For instance, Pseudomonas aeruginosa uses its Type III secretion system (T3SS) to inject effector cytotoxins (ExoS, ExoT, ExoU) into host immune cells to disrupt actin cytoskeletons, while simultaneously deploying its Type II secretion system (T2SS) to export soluble elastase (LasB) and exotoxin A to degrade tissue barriers. Parallel mechanisms drive Gram-positive virulence; Staphylococcus aureus secretes a diverse arsenal of leaderless alpha- and beta-hemolysins via unconventional routes alongside classical signal-peptide-dependent enterotoxins [75,76]. The bacterial secretome plays critical roles in immune evasion, biofilm formation, and establishing infection niches. Horizontal gene transfer frequently mobilizes secretome-encoding genes, accelerating the evolution of cooperative and virulent traits. Beyond classical protein secretion, bacterial extracellular vesicles further modulate host responses and microbial community dynamics [75,76].

In parallel, filamentous fungi are among the most prolific secretors in the biosphere. Fungal secretomes are dominated by carbohydrate-active enzymes (CAZymes), proteases, lipases, and secondary metabolites that allow them to function as saprotrophs, pathogens, or symbionts. In pathogenic fungi, secreted effectors and enzymes facilitate host invasion, immune suppression, and nutrient extraction.

For example, the industrial workhorse *Trichoderma reesei* upregulates and secretes large amounts of cellobiohydrolases (Cel7A and Cel6A) and endoglucanases when triggered by cellulose breakdown products. This cascade is heavily regulated by the transcription factors XYR1 and CRE1. In pathogenic fungi such as Magnaporthe oryzae (rice blast), specialized unconventional secretion pathways bypass classical Golgi routing to deliver leaderless effector proteins (e.g., Avr-Piz-t) directly into the host apoplast via specialized structures called biotrophic interfacial complexes (BICs), establishing a molecular arms race for host immune suppression [8,11,77]. Conversely, in mutualistic interactions, the secretome mediates nutrient exchange and host colonization. Industrially, filamentous fungi’s high secretory capacity has been harnessed. Species such as *Trichoderma reesei*, *Aspergillus*, and *Penicillium* produce complex enzyme cocktails used in biofuel production, food processing, textile treatment, and bioremediation. Modern secretomics, combining genome mining with high-throughput proteomics, has accelerated the discovery and bioengineering of optimized fungal enzyme cocktails. At the same time, synthetic biology approaches continue to expand the industrial repertoire to include heterologous proteins and complex secondary metabolites [2,77,78].

### 4.4. Plant Secretomes: Apoplast, Defense, Root Exudates, Unconventional Secretion

Unlike mammalian cells, plant cells have a rigid cell wall and release secretory products into the apoplast. This continuous extracellular space is a major site of cell-to-cell communication, nutrient exchange, structural support, and defense. The plant secretome includes soluble proteins, peptides, metabolites, extracellular vesicles, and cell-wall-modifying enzymes that shape local tissue environments and the broader rhizosphere. The apoplast depends on a carefully orchestrated influx of components to support development and stress survival. Canonical secretion via the endoplasmic reticulum–Golgi pathway delivers many cell-wall proteins, enzymes, and signaling peptides [2,79]. However, many experimentally detected apoplastic proteins lack canonical signal peptides, indicating the importance of unconventional protein secretion (UPS) routes in plants [80]. These leaderless secreted proteins can make up more than half of the total secretome under specific environmental regimes. This figure is now widely recognized as an overestimate because standard apoplastic extraction workflows are highly susceptible to cytoplasmic contamination from minor cellular rupture, which introduces abundant intracellular passengers into the secretome dataset. Early computational validations relied heavily on mammalian-trained prediction tools like SecretomeP, which show false-positive rates exceeding 30% when applied to plants. Thus, while UPS exists, many “leaderless” detections are now attributed to contamination or cell damage rather than true UPS, so the quantitative importance is overstated compared to rigorous contamination-controlled studies [35].

Upon pathogen attack or herbivory, plants rapidly reprogram their secretome output to secure the extracellular space. They secrete pathogenesis-related (PR) proteins, proteases, chitinases, glucanases, and antimicrobial peptides into the apoplast or at infection sites. Extracellular vesicles also contribute to defense by delivering small RNAs, proteins, and metabolites that inhibit pathogen growth or modulate host immune responses. Vacuole–plasma membrane fusion and other unconventional routes can release stored defense compounds during intensive stress responses [81,82]. Mechanistically, recognition of fungal pathogen-associated molecular patterns (PAMPs) triggers a rapid influx of calcium ions and a burst of ROS that shifts apoplastic pH. This microenvironmental change drives massive transcriptional upregulation and classical exocytic secretion of specialized pathogenesis-related (PR) proteins. Among these, PR-2 (beta-1,3-glucanases) and PR-3/PR-4 (chitinases) are directionally deployed to the infection interface. These enzymes coordinate to hydrolyze the structural beta-1,4-linkages of invading fungal cell walls, degrading hyphae while releasing chitin elicitor fragments that amplify host defense signaling [82].

Concurrently, plants bypass cell wall barriers against fungal pathogens by mobilizing multivesicular bodies (MVBs) to release defense-adapted plant EVs into the apoplastic fluid. These EVs serve as protected, mobile units that encapsulate lipophilic secondary metabolites, defense proteins, and host-derived small RNAs (sRNAs). Once in the apoplast, these vesicles undergo trans-kingdom trafficking, transferring host sRNAs directly into invading fungal cells to initiate cross-kingdom RNA interference (RNAi). This process selectively silences essential fungal virulence and housekeeping genes, illustrating a specialized, multi-component defense mechanism operating natively within the plant apoplast [11,82]. Vacuole–plasma membrane fusion and other unconventional routes can release stored defense compounds during intensive stress responses [81,82].

Below-ground root systems continuously synthesize and release a vast molecular cocktail into the adjacent soil matrix called root exudates. These compounds shape the rhizosphere microbiome, mobilize nutrients (e.g., via phosphatases and proteases), deter pathogens, and facilitate symbiotic interactions with beneficial microbes such as mycorrhizal fungi or nitrogen-fixing bacteria. Recent work highlights their roles in nutrient acquisition, microbial recruitment, and plant defense, involving both classical and unconventional secretion pathways, including multivesicular bodies and specialized organelles [83,84]. However, methodological challenges such as cytoplasmic leakage during apoplastic fluid extraction, limitations of mammalian-trained prediction models, and the technical complexity of root-exudate isolation require dedicated plant-specific experimental and computational approaches.

### 4.5. Comparative Insights: Conserved Mechanisms vs. System-Specific Features

A systematic comparison of secretomes across mammalian, stem-cell, microbial, fungal, and plant systems reveals a consistent evolutionary pattern. Deeply conserved core principles coexist with highly specialized, lineage-specific innovations. Classical and unconventional routes in eukaryotes operate alongside specialized ones in bacteria. Leaderless proteins reach the extracellular space through unconventional pathways such as membrane budding, multivesicular bodies, specialized organelles, or direct translocation [85]. Extracellular vesicles function as versatile carriers of proteins, nucleic acids, lipids, and metabolites in every major group examined. Biogenesis details differ, but packaging and delivering complex cargo in membrane-bound particles is a broadly conserved mode of extracellular communication [21,86,87]. Secretomes are molecularly heterogeneous and plastic. Source, physiological state, and preconditioning strongly shape both the soluble and vesicular fractions, from MSCs to bacterial pathogens to plant root cells [74,81,83].

**Figure 2:**
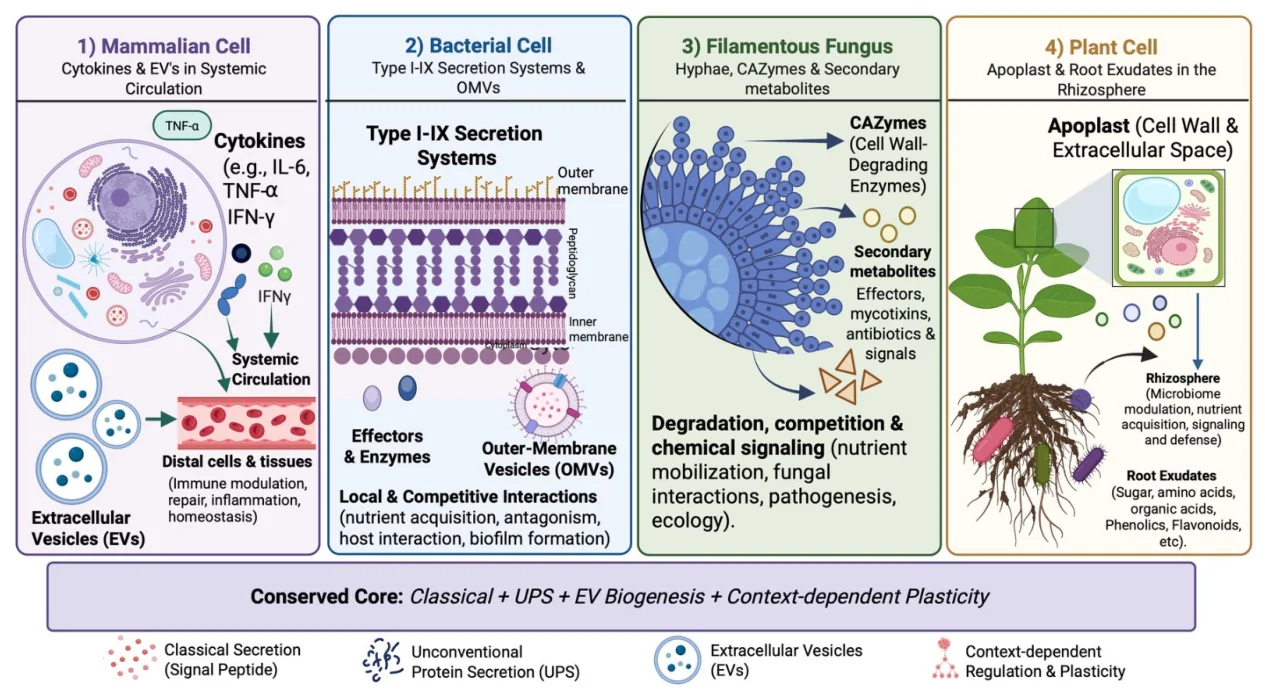
Comparative cross-domain secretomics and conserved secretory architectures. Comparative analysis of cross-domain secretomics and conserved secretory architectures. The illustration presents distinct secretome profiles across biological kingdoms, highlighting a conserved core comprising classical secretion, unconventional protein secretion (UPS), extracellular vesicle (EV) biogenesis, and context-dependent plasticity. (1) Mammalian cells secrete cytokines (e.g., IL-6, TNF-α, IFN-γ) and EVs into systemic circulation to regulate distal cell and tissue signaling, including immune modulation, tissue repair, inflammation, and homeostasis. (2) Bacterial cells employ Type I to Type IX secretion systems and outer-membrane vesicles (OMVs) for local and competitive interactions, such as nutrient acquisition, antagonism, host interaction, and biofilm formation. (3) Filamentous fungi release carbohydrate-active enzymes (CAZymes) and secondary metabolites via hyphae to support degradation, competition, chemical signaling, and pathogenesis. (4) Plant cells secrete factors into the apoplast (cell wall and extracellular space) and release root exudates (sugars, amino acids, organic acids, phenolics, flavonoids) to influence the rhizosphere microbiome, nutrient acquisition, and defense.

Alongside conservation, each system exhibits distinctive innovations. Stem and progenitor cells have specialized secretomes optimized for niche maintenance, tissue repair, and immunomodulation. Bacteria deploy an extraordinary diversity of dedicated secretion systems that function as molecular syringes for virulence effectors, toxins, and competitive weapons [88]. Fungi, especially filamentous species, are exceptional secretors of hydrolytic enzyme cocktails and secondary metabolites [89]. Plants direct the secretion primarily into the apoplast and the rhizosphere [79,80]. Table 2 summarizes conserved mechanisms and system-specific features of secretomes across biological kingdoms, while **Figure 2** illustrates the corresponding cross-domain secretome landscapes.

Mammalian cells release systemic cytokines and extracellular vesicles (EVs) to mediate distal tissue signaling and maintain homeostasis. Bacterial cells utilize Type I to Type IX secretion systems and outer membrane vesicles (OMVs) for local competition and biofilm formation. Filamentous fungi secrete CAZymes and secondary metabolites through hyphae to facilitate biomass degradation and pathogenesis. Plant cells deploy apoplastic factors and root exudates to modulate the rhizosphere microbiome.

## 5. Applications and Translational Landscape

### 5.1. Diagnostics and Biomarkers: The Circulating Secretome as a Noninvasive Diagnostic Window

#### 5.1.1. Liquid Biopsy Paradigms in Oncology

The circulating secretome provides a real-time, minimally invasive window into tissue physiology and pathology. This approach is especially promising in cancer. Tumor-derived EVs and secreted factors carry molecular fingerprints of the cell of origin. This enables applications in early detection, diagnosis, prognosis, and therapy monitoring [90,91]. Preclinically, EVs are attractive liquid-biopsy analytes. They protect their cargo (proteins, mRNAs, miRNAs, DNA fragments, metabolites) from degradation. They are present at relatively high concentrations in blood, urine, saliva, cerebrospinal fluid, and other accessible fluids. They also reflect both the identity and physiological state of the releasing cells [92,93]. Recent technological advances include single-EV analysis, multi-omics profiling, CRISPR-based amplification of rare EV transcripts, and artificial-intelligence-driven signature discovery. These may further improve analytical sensitivity and specificity [94,95].

#### 5.1.2. Non-Oncological Manifestations and Multi-Omic Fingerprints

Researchers are exploring secretome-derived biomarkers in neurodegenerative diseases, cardiovascular disease, chronic inflammation, fibrosis, and metabolic disorders. Disease-specific secretory signatures—whether protein panels, EV surface markers, or multicomponent “secretomic fingerprints”—offer earlier diagnosis and more precise patient stratification than single-analyte assays [96]. The diagnostic value of the circulating secretome is not limited to stem-cell or cancer-cell products. Hepatocytes, adipocytes, endothelial cells, immune cells, and many other populations contribute distinct, dynamically regulated proteins to the plasma proteome. Large-scale, multicenter validation studies and integrating multi-omics data with machine-learning algorithms will be essential to translate promising secretome biomarkers into routine clinical practice [97,98,99].

### 5.2. Cell-Free Therapeutics and Regenerative Applications: Toward Next-Generation Biologics

#### 5.2.1. The Paracrine Shift in Regenerative Medicine

Recognizing that the therapeutic benefits of stem-cell transplantation are largely mediated by paracrine factors, rather than direct cellular engraftment and differentiation, has driven a major shift toward cell-free regenerative strategies [100]. Secretome-based products are being investigated as off-the-shelf biologics that retain the regenerative, immunomodulatory, anti-inflammatory, pro-angiogenic, and anti-fibrotic activities of their parental cells while offering practical advantages in safety, scalability, storage, and standardization [64,101].

#### 5.2.2. The Mesenchymal Stem Cell Milestone and Bio-Priming Strategies

Preclinical studies of MSC-derived secretomes and EVs in models of wound healing, myocardial infarction, ischemic stroke, osteoarthritis, lung injury, liver fibrosis, and neurodegenerative disease have reported improvements in tissue repair, inflammatory responses, and functional outcomes. These effects come from a complex mixture of growth factors (e.g., VEGF, HGF, IGF-1, TGF-β), cytokines, chemokines, matrix-modulating enzymes, and EV-packaged miRNAs and proteins. Together, they promote angiogenesis, cell survival, macrophage polarization toward reparative phenotypes, and fibrosis resolution. Preconditioning strategies such as hypoxia, inflammatory cytokines, 3D culture, or pharmacological priming may enhance bioactivity and allow tailoring of secretome composition for specific applications [102,103,104,105,106,107].

#### 5.2.3. Expanding to Non-MSC and Differentiated Cell Lineages

Secretomes or EVs from induced pluripotent stem cell–derived mesenchymal progenitors, neural stem/progenitor cells, endothelial progenitors, cardiac progenitors, and certain tissue-resident stem cells exhibit tissue-specific regenerative activities [108,109]. In some cases, these non-MSC sources offer complementary bioactivity, reduced donor variability, or better matching to the target tissue niche [110,111,112]. Beyond stem cells, researchers are increasingly exploring secretomes from other mammalian cell types, such as macrophages, fibroblasts under controlled conditions, or engineered cells, for specialized regenerative applications [113,114]. For example, engineered exosome platforms using stem cell-derived vesicles can be loaded with specific antioxidant cargo such as epigallocatechin-3-gallate (EGCG). This cargo suppresses pro-inflammatory cytokine cascades (e.g., IL-6, NF-κB) and regulates apoptotic pathways (e.g., Bax, Bcl-2) in target tissues [115]. These approaches highlight the utility of tailoring secretome composition to achieve precise, targeted therapeutic mechanisms in inflammatory disease models.

#### 5.2.4. Overcoming Translational Bottlenecks and Clinical Gaps

Cell-free secretomic approaches may reduce several liabilities of live-cell therapies, including risks of ectopic tissue differentiation and tumor formation, while also easing logistical challenges of cryogenic live-cell storage and transport. Secretome-based products can be stabilized through lyophilization or cryopreservation, facilitating storage and distribution [116]. However, clinical translation remains constrained by pronounced batch heterogeneity from varying donor tissues, culture passage limits, and distinct physical isolation workflows. The field also lacks robust, mechanism-based potency assays that accurately predict in vivo clinical efficacy [117].

Current clinical activity remains investigative, mainly focused on early-phase trials evaluating MSC secretome or EV preparations for wound healing, osteoarthritis, cardiovascular disease, and neurological indications [118,119]. Parallel efforts explore engineered secretomes—genetically modified cells overexpressing therapeutic factors or EVs loaded with drugs, nucleic acids, or targeting ligands—to increase potency and specificity [120,121]. Implementing conceptual and analytical advances that enable standardized secretome mapping across mammalian, plant, and microbial domains may facilitate development of more predictable, scalable, and standardized regenerative products.

### 5.3. Anticancer Potential and Immune Modulation: Resolving Conflicting Data via Structured Decision Frameworks

#### 5.3.1. The Dualistic Secretomic Paradox in Oncology

The secretome plays a highly context-dependent role in cancer biology and therapeutics. Secretory programs from tumor cells, cancer-associated fibroblasts (CAFs), and myeloid-derived suppressor cells (MDSCs) can promote malignant progression, angiogenesis, extracellular matrix (ECM) remodeling, and immune evasion in the tumor microenvironment (TME). Conversely, secretome preparations from molecularly primed or genetically engineered MSCs and other progenitor populations have shown antitumor activity in selected preclinical settings, including inhibiting tumor growth, modulating angiogenic signaling, inducing tumor-cell death, and altering immune responses [122,123,124]. This duality highlights the context-dependent nature of secretome bioactivity and the need for multiparametric interpretation before clinical translation.

#### 5.3.2. Biological and Technical Determinants of Secretomic Bioactivity

Multiple biological and technical variables can influence whether a given secretome preparation exhibits pro- or antitumorigenic properties. These include:

(i) Ontogenetic Source and Lineage: Secretomes derived from perinatal MSC sources, such as umbilical cord or Wharton’s jelly, may differ functionally from those obtained from adult bone marrow or adipose tissue. Tissue origin can influence secretome composition, regenerative signaling, and potential antitumor activity.

(ii) Culture Conditions and Bio-Priming: Serum composition, inflammatory priming (e.g., IFN-γ, TNF-α, or IL-1β), and hypoxic preconditioning can modify secretome composition and the levels of factors linked to angiostatic, inflammatory, or pro-apoptotic responses, including TRAIL, DKK-1, and CXCL10. Conversely, unprimed or serum-containing conditions may introduce growth-promoting signals or confounding serum-derived factors. These may contribute to variable oncological outcomes.

(iii) Biophysical Fractions (EVs vs. Soluble Proteome): Extracellular vesicles and soluble secretome fractions can contain distinct and sometimes opposing bioactive components. EV cargo may include pro-survival or pro-angiogenic factors, whereas other EV populations can carry tumor-suppressive molecules. Accordingly, separating and independently characterizing vesicular and soluble fractions is necessary to determine their respective contributions to overall bioactivity.

(iv) TME Architecture and Tumor Models: Secretome bioactivity can vary across cancer lineages, mutational profiles, disease stages, and host immune states. Experimental outcomes may also differ according to tumor model, treatment timing, and whether the intervention is evaluated in preventive or therapeutic settings.

(v) Dosing and Pharmacokinetic Variables: Differences in dose, administration frequency, treatment duration, and delivery route, including systemic and intratumoral administration, may cause discrepancies among studies. Standardized dosing and pharmacokinetic characterization are necessary for meaningful cross-study comparison.

**Figure 3:**
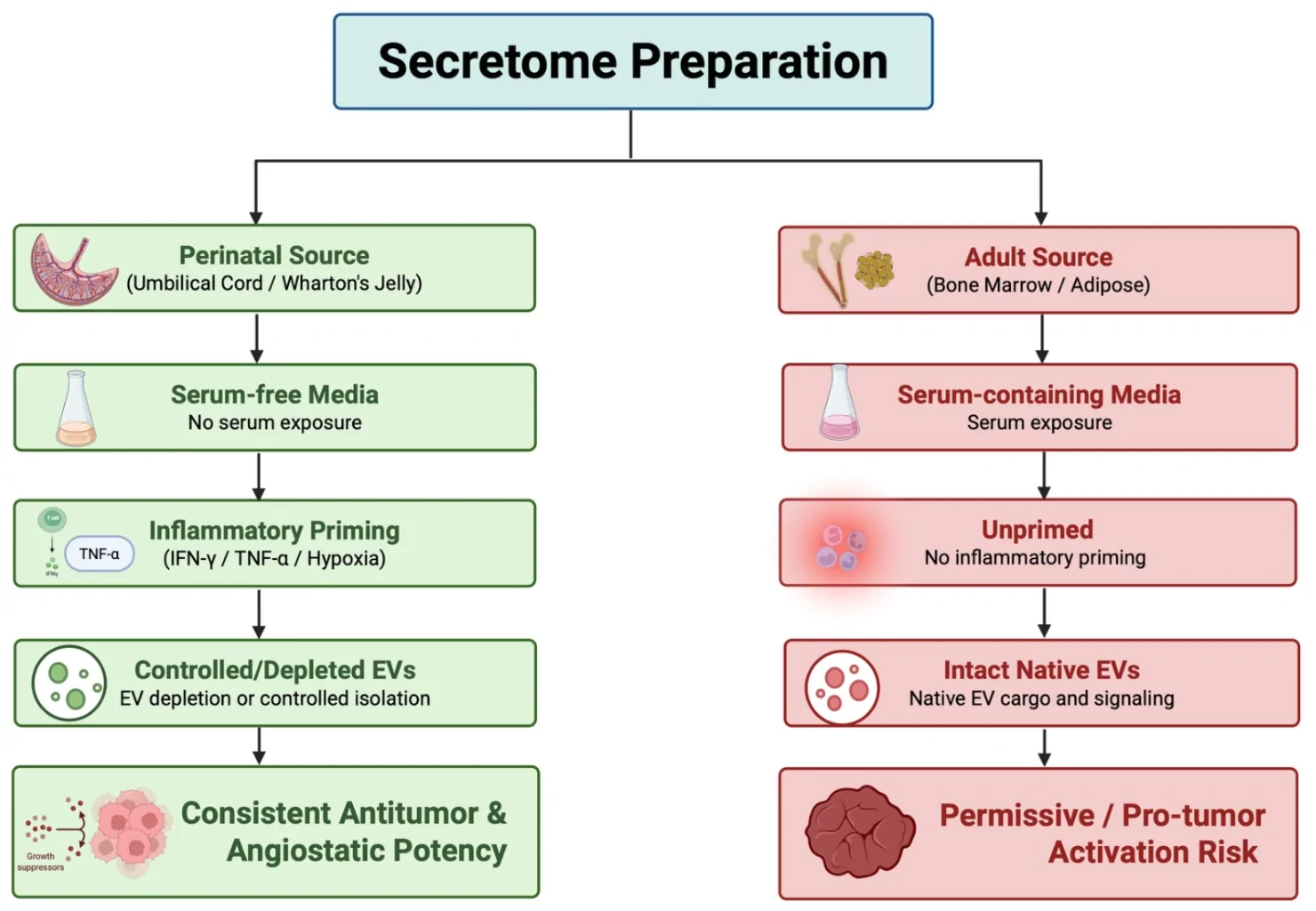
Methodological optimization workflows for secretome preparation and therapeutic outcome stratification. Methodological optimization workflows for secretome preparation and therapeutic outcome stratification. Precise control of upstream and downstream production parameters determines secretome bioactivity, analytical accuracy, and functional outcomes. Perinatal sources (umbilical cord, Wharton’s jelly) cultured in serum-free media with specific inflammatory priming (IFN-γ, TNF-α, or hypoxia) and controlled or depleted EV isolation protocols consistently yield antitumor and angiostatic effects. In contrast, adult sources (bone marrow, adipose tissue) maintained in serum-containing media without inflammatory priming and using intact native EVs carry a higher risk of permissive or pro-tumor activation. Systematic control, characterization, and reporting of these variables are essential for reproducible cross-study comparisons.

Taken together, available evidence suggests that cell source, culture conditions, priming strategy, and the relative contribution of soluble and vesicular fractions can substantially influence oncological effects. A systematic review of 21 clinical reports has found that EV therapy is generally safe, with low rates of serious adverse events (0.7%) and adverse events (4.4%). No clear safety differences were observed between autologous and allogeneic products or between engineered and native products. Some patients receiving immunostimulatory, immunosuppressive, or regenerative EV treatments showed signs of improvement, but no definitive evidence of clinical benefit has been shown. In addition, heterogeneity in the manufacturing processes, study design, and adverse event reporting limits the evidence [125]. Figure 3 summarizes these methodological variables and their potential influence on therapeutic outcome.

Secretome bioactivity can be influenced by cell source, culture medium, priming conditions, and the relative contribution of extracellular vesicle (EV) and soluble fractions. Perinatal or adult cell sources, serum-free or serum-containing conditions, inflammatory or hypoxic priming, and EV isolation or depletion may each alter secretome composition and downstream antitumor or protumor effects. Consequently, these variables should be systematically controlled, characterized, and reported to enable reproducible comparison across experimental models.

#### 5.3.3. A Standardized Decision Framework for Predictive Bioengineering

A structured decision framework may help improve comparability and predictive bioengineering. We propose six core considerations:

**(i) Define the Intended Therapeutic Mechanism:** Clearly isolate the primary intended biological mechanism, such as direct tumor cytotoxicity, anti-angiogenesis, immune microenvironment re-education, or stromal reprogramming.

**(ii) Standardize and Document Critical Experimental Variables:** Quantify Critical Quality Attributes (CQAs) such as donor demographics, priming regimens, passage numbers, and serum status of the culture medium.

**(iii) Use Orthogonal Functional Assays:** Validate secretome batches with multi-tiered assays capturing proliferation kinetics, migration/invasion dynamics, endothelial tube formation, and macrophage polarization.

**(iv) Resolve Contributions of Secretome Fractions:** Experimentally separate contributions of the soluble proteome and vesicular fractions using high-resolution ultracentrifugation or size-exclusion chromatography.

**(v) Select Biologically Appropriate In Vivo Models:** Use models suited to the biological mechanism and disease context. Immunocompetent syngeneic models are informative for immune-mediated effects. Humanized models and patient-derived xenografts (PDXs) provide complementary insights into human tumor biology and microenvironment interactions.

**(vi) Develop Multiparametric Potency Metrics:** Move beyond crude total protein concentration readouts by establishing standardized potency metrics linked to specific bioactive analytes or functional cell responses.

By incorporating this framework, researchers can systematically benchmark studies and rationally design next-generation anticancer biologics. Advanced engineered approaches such as loading TRAIL, interferons, or tumor-suppressive miRNAs into targeted EVs or combining pharmacological priming with controlled EV cargo engineering may enhance antitumor activity while reducing unintended protumorigenic effects.

#### 5.3.4. Reprogramming the Tumor Immune Microenvironment (TIME)

Beyond direct effects on tumor cells, secretome components can modulate the tumor immune microenvironment (TIME) by altering macrophage functional states, T-cell activity, and local cytokine networks. Depending on secretome composition and disease context, these effects may enhance or suppress antitumor immunity. However, whether the net immunological outcome is immuno-stimulatory or immunosuppressive is strongly influenced by the production variables and contextual parameters outlined above. High-resolution mapping of secretome composition to downstream immune phenotypes is therefore important for defining therapeutic activity and identifying potential immunosuppressive effects [106]. Such characterization is relevant not only for oncological applications but also when secretome-based products are considered in regenerative or chronic inflammatory settings.

### 5.4. Industrial and Agricultural Applications: Leveraging Cross-Kingdom Secretory Capacity

Beyond mammalian biomedicine, secretomes derived from microbes, fungi, and plants underpin major industrial and agricultural technologies. These large-scale applications leverage the natural secretory capacity of non-mammalian systems, illustrating the practical value of establishing a unified, cross-domain understanding of extracellular export.

#### 5.4.1. Industrial Enzyme Production and Microbial Biotechnology

Modern fungal secretomics—combining genome mining, proteomics, and systems biology—has accelerated the discovery of novel enzymes and the engineering of engineered secretion strains. Synthetic biology approaches further expand the repertoire by enabling fungi to secrete heterologous enzymes or multi-enzyme complexes at high titers [126]. Bacterial secretion systems are likewise exploited for the recombinant production of industrial enzymes, therapeutic proteins, and specialty chemicals. Engineering of Type II, Type III, and other secretion pathways, together with extracellular-vesicle platforms, is being explored to improve production yields and product characteristics [127,128].

#### 5.4.2. Sustainable Agriculture, Root Exudates, and Biocontrol Agents

Plant and microbial secretomes also play central roles in sustainable agriculture. Root exudates shape rhizosphere microbiomes, mobilize soil nutrients, suppress pathogens, and promote beneficial symbioses with mycorrhizal fungi and nitrogen-fixing bacteria. Understanding and engineering these secretory programs may provide strategies to reduce fertilizer and pesticide inputs, enhance crop resilience, and improve soil health. High-throughput secretome profiling is increasingly being applied to identify and characterize bioactive components with potential agricultural applications. In parallel, plant-derived extracellular vesicles and defense-related secreted proteins are being explored as natural or bioengineered biostimulants and bioactive agents for crop protection [129,130].

#### 5.4.3. Cross-Domain Synergy and Bioproduct Design

Comparative secretome knowledge creates new translational possibilities. Methodologies refined in mammalian systems (metabolic labeling, advanced mass spectrometry, multi-omics integration) can improve the characterization of industrial fungal and bacterial secretomes. Conversely, the high secretory capacity and robustness of microbial systems may provide useful design principles and biological chassis for engineered production systems. Insights into unconventional secretion and extracellular-vesicle biogenesis across kingdoms may also inspire novel delivery or encapsulation technologies for agricultural and industrial products [131,132]. Industrial and agricultural applications face distinct standardization and regulatory challenges, including batch-to-batch consistency of enzyme preparations, environmental safety of engineered microorganisms, and the need for cost-effective and scalable production. From an economic perspective, while fungal secretome production for industrial enzymes operates at high scales, the downstream isolation and concentration of specialized proteins remain highly cost-intensive, often making up over 40% of total bioprocess manufacturing outlays. Economically viable adoption in agriculture requires raw root exudate or bio-stimulant formulations to achieve low cost-per-acre metrics to successfully compete with traditional synthetics. In addition, key environmental safety hurdles revolve around the biocontainment of genetically engineered microbial strains deployed as agricultural soil inoculants. Biocontainment strategies, including synthetic auxotrophy and genomic suicide circuits, are heavily scrutinized to mitigate the risk of horizontal gene transfer to wild soil microbiomes. Mechanistically, these commercial barriers are highly polarized by regional regulatory frameworks: the European Union operates under strict, hazard-based precautionary principles via the European Food Safety Authority (EFSA), requiring intensive long-term environmental impact profiles for microbial lysates, whereas the United States EPA and FDA utilize more permissive, risk-based frameworks that grant Generally Recognized as Safe (GRAS) statuses far more rapidly for optimized industrial secretome extracts. Nevertheless, secretome research provides an important interface between fundamental cell biology and applied biotechnology.

### 5.5. Standardization, GMP Production, Delivery Strategies, and Regulatory Considerations

The translational development of secretome-based products—whether intended as diagnostic tools, cell-free regenerative therapeutics, anticancer formulations, or industrial bioproducts—requires robust standardization, scalable manufacturing, appropriate delivery strategies, and regulatory frameworks adapted to the intended application. These requirements remain under development and represent important challenges for clinical and commercial translation.

#### 5.5.1. Defining Critical Quality Attributes (CQAs) and Potency Metrics

The inherent complexity of secretomes makes defining critical quality attributes (CQAs) like identity, purity, potency, safety, and stability more challenging than for single-molecule products. For example, current investigational good manufacturing practice (GMP) workflows for MSC-derived extracellular vesicles use hollow-fiber bioreactors for scalable, continuous conditioning under chemically defined, animal-component-free conditions. Downstream purification involves sequential tangential flow filtration (TFF) and size-exclusion chromatography (SEC), with explicit parameters for CQAs. These include verifying identity through Western blot or flow cytometry tracking of tetraspanins (CD63+/CD81+/CD9+), ensuring purity by enforcing threshold limits for residual host–cell DNA (<10 ng/dose) and endotoxins (<5 EU/kg), and assessing batch potency using standardized in vitro target cellular assays such as real-time endothelial cell tube formation metrics.

Key areas requiring standardization include:

**(i) Standardized Upstream Protocols:** Developing well-defined and reproducible procedures for cell expansion, serum-free or chemically defined culture conditions, and secretome/EV collection.

**(ii) Orthogonal Characterization Strategies:** Combining complementary analytical approaches, including quantitative proteomics, nanoparticle tracking analysis, and multi-omics profiling, to characterize composition and assess batch-to-batch consistency.

**(iii) Mechanism-Based Potency Assays:** Developing functional assays linked to the intended biological mechanism of action, rather than relying solely on total protein concentration or particle counts.

**(iv) Validated Reference Materials:** Developing appropriate reference materials and organizing interlaboratory comparison studies to improve reproducibility across datasets and laboratories.

International societies and multi-institutional consortia focused on extracellular vesicles and cell-free biologics continue to refine reporting and characterization guidelines [133,134]. Similar principles may provide a useful foundation for broader standardization across secretome research. Table 3 summarizes key quality attributes and corresponding analytical approaches.

#### 5.5.2. GMP-Compliant Biomanufacturing and Downstream Processing

Transitioning secretome products to clinical-grade manufacturing requires scalable, well-controlled production systems and reproducible upstream and downstream processes. Using chemically defined and animal-component-free media, where appropriate, can minimize variability and support product characterization. Validated downstream processes may enrich or deplete specific secretome fractions, including extracellular vesicles (EVs). Quality-control strategies should include testing for relevant contaminants and impurities such as adventitious agents, bacterial endotoxins, and residual host–cell proteins or nucleic acids, as appropriate for the product [134,135]. Because storage conditions affect the stability and biological activity of secretome components, current research is investigating lyophilization and other stabilization methods to enhance shelf life and support distribution [136,137]. Notably, microbial and fungal industrial enzyme production is already conducted at large scale under established GMP or equivalent frameworks, which may provide relevant manufacturing principles for mammalian secretome production.

#### 5.5.3. Pharmacokinetics and Targeted Delivery Strategies

The delivery route and formulation significantly affect tissue distribution, retention, and pharmacokinetic properties of secretome-based products. Several delivery strategies are currently being evaluated in preclinical and clinical studies, including:

**(i) Systemic Administration:** Intravenous administration provides systemic exposure but may be constrained by rapid uptake or clearance in organs such as the liver and spleen. Researchers are investigating surface engineering of EVs with targeting ligands to modify biodistribution.

**(ii) Localized Delivery:** Direct injection or implantation at the target tissue, such as cutaneous wounds. (iii) **Biomaterial-Based Controlled Release:** Encapsulation of soluble factors or vesicular fractions within biocompatible hydrogels, structural scaffolds, or specialized slow-release biomaterials to extend local therapeutic duration.

Accordingly, the route, dose, administration schedule, and formulation should be optimized based on the intended application [138,139].

#### 5.5.4. Global Regulatory Classifications and In Vitro Diagnostic (IVD) Guidelines

Regulatory classification of secretome products varies significantly across jurisdictions. It depends mainly on the intended clinical or commercial application and is influenced by factors such as product composition, manufacturing process, mechanism of action, and intended use. Mammalian cell-derived secretomes and extracellular vesicle (EV)-based therapeutics may be regulated under frameworks for biological or advanced medicinal products, depending on jurisdiction and specific product characteristics. Therefore, requirements for manufacturing quality, nonclinical safety, and clinical evaluation must be tailored to each product. In the United States, the Food and Drug Administration (FDA) classifies native mammalian-cell-conditioned media and isolated EV therapeutics as Human Cells, Tissues, and Cellular and Tissue-Based Products (HCT/Ps) under Section 351 of the Public Health Service Act, requiring full Investigational New Drug (IND) approval. In contrast, the European Medicines Agency (EMA) generally regulates these products under the Advanced Therapy Medicinal Products (ATMP) framework or as biological medicinal products, depending on whether the cellular origin is entirely acellular.

**Figure 4:**
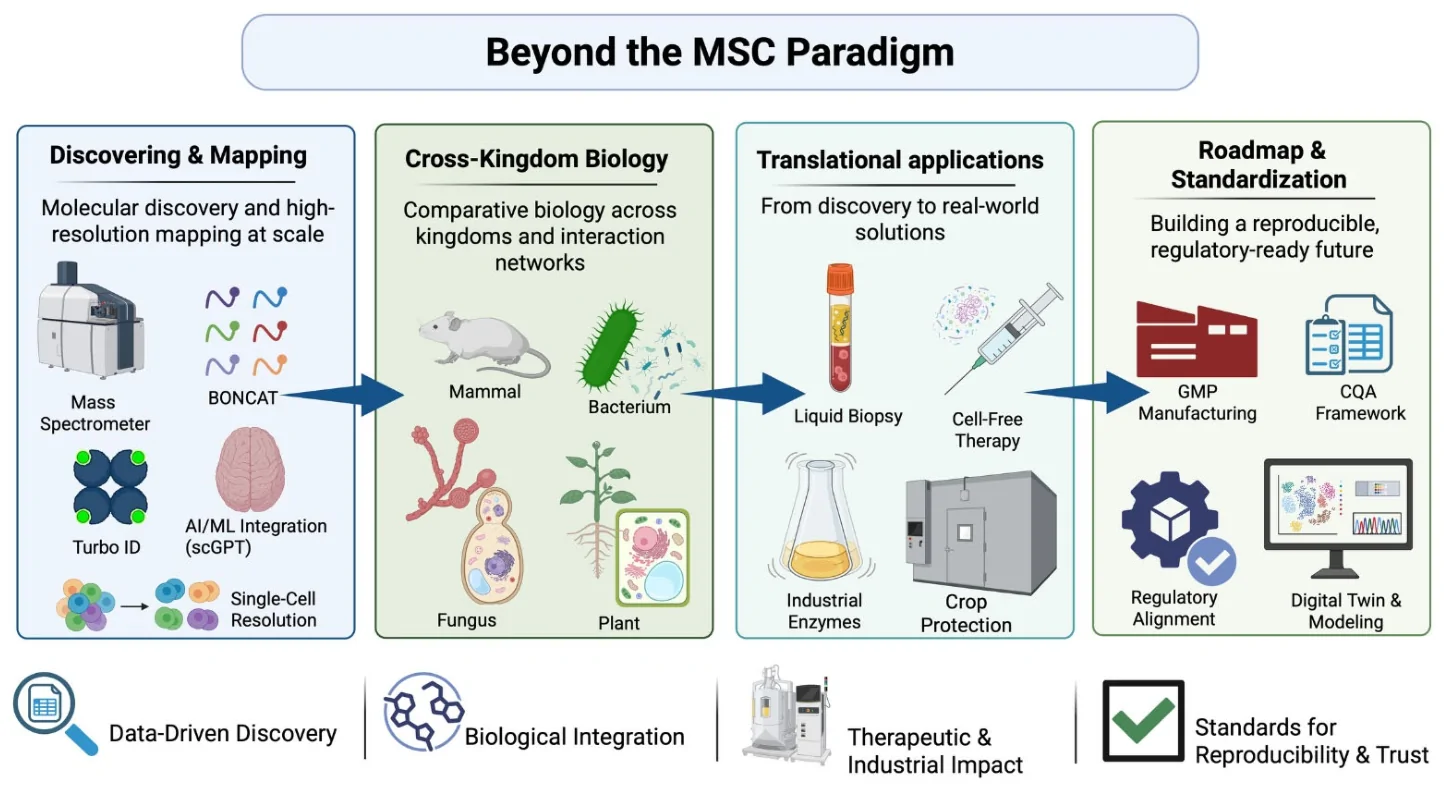
Conceptual framework for high-resolution, cross-kingdom secretomics and translational standardization. Conceptual framework for high-resolution, cross-kingdom secretomics and translational standardization. The proposed pipeline advances beyond the traditional mesenchymal stromal/stem cell (MSC) paradigm through four sequential phases. (1) Discovery and Mapping uses mass spectrometry, bioorthogonal non-canonical amino acid tagging (BONCAT), TurboID, single-cell resolution, and artificial intelligence or machine learning integration (scGPT) for high-resolution molecular identification. (2) Cross-Kingdom Biology establishes comparative networks across mammalian, bacterial, fungal, and plant systems. (3) Translational Applications implement identified targets in practical solutions such as liquid biopsy, cell-free therapy, industrial enzymes, and crop protection. (4) Roadmap and Standardization ensure reproducibility and regulatory readiness through Good Manufacturing Practice (GMP) compliance, Critical Quality Attribute (CQA) frameworks, regulatory alignment, and digital twin modeling.

Engineered exosome platforms that deliver exogenous molecular cargo, such as the EGCG antioxidant loading model, are increasingly reviewed under specialized drug-delivery or combination biologic/drug regulatory frameworks. These frameworks require separate chemical, manufacturing, and control (CMC) validations for both the vesicle carrier and the therapeutic cargo. Secretome-derived biomarkers intended for diagnostic applications are typically regulated within in vitro diagnostic (IVD) frameworks. Industrial enzymes and agricultural biocontrol products follow distinct regulatory pathways based on their intended use, manufacturing process, and potential environmental impact. Because of this regulatory complexity, early engagement with relevant regulatory authorities is recommended to clarify product classification and development requirements, especially as guidance for complex cell-free and EV-based products continues to evolve. Figure 4 presents a proposed analytical-to-translational pipeline.

High-resolution discovery and mapping integrate mass spectrometry, proximity-labeling approaches, single-cell platforms, and computational analysis across mammalian, bacterial, fungal, and plant systems. Validated molecular signatures can subsequently inform diagnostic, therapeutic, industrial, and agricultural applications. Translational development requires standardized analytical workflows, good manufacturing practice (GMP) where applicable, critical quality attribute (CQA) frameworks, and regulatory alignment.

## 6. Emerging Frontiers and Future Directions

Advancing and clinically translating secretome-based approaches increasingly relies on precise characterization, control, and modeling of complex molecular communication networks. Beyond standardizing native cell cultures and secretome preparations, recent strategies move the field from descriptive profiling toward rational, high-resolution analysis and design. Developments in synthetic biology, spatial and single-cell technologies, and artificial intelligence enhance the ability to interrogate and engineer secretory networks. The following section outlines technological innovations that may facilitate the transition from descriptive secretome catalogs to predictive models of intercellular and inter-organ communication.

### 6.1. Engineered and Primed Secretomes

A significant emerging direction in secretome research focuses on engineering or priming cells to alter the composition and biological activity of their secretory output. Instead of relying solely on the native secretome of specific cell types, researchers are increasingly investigating strategies to customize secretome composition for therapeutic applications [140,141].

Priming, or preconditioning, is one of the most accessible strategies for modifying secretome composition. Exposing mesenchymal stem cells (MSCs) or other progenitor cells to hypoxia, inflammatory cytokines such as IFN-γ or TNF-α, three-dimensional (3D) culture, pharmacological agents, genetic modifications, or disease-relevant microenvironmental cues can alter both soluble and vesicular secretome fractions [142,143]. Inflammatory and hypoxic preconditioning link to changes in immunomodulatory, chemotactic, angiogenic, and cytoprotective components; however, the resulting biological activity depends on cell source and experimental conditions. While these approaches may enhance specific functional properties, they can also introduce batch variability that requires careful control [144,145].

Genetic and molecular engineering approaches offer more direct control over secretome composition. Cells may be engineered to overexpress therapeutic proteins, including TRAIL, specific growth factors, or anti-inflammatory cytokines; to enrich extracellular vesicles (EVs) with selected microRNAs (miRNAs), messenger RNAs (mRNAs), or CRISPR-associated components; or to display targeting ligands on EV surfaces [146,147]. Synthetic biology circuits that sense environmental signals and modulate secretory output are also in development [23]. Additionally, researchers are exploring strategies to manipulate EV biogenesis to alter vesicle yield, cargo composition, or targeting properties [148].

Hybrid and multisource strategies are also under investigation. Combining secretome fractions from various cell types or integrating soluble factors with engineered EVs can generate complementary functional profiles [149]. In industrial and agricultural contexts, engineering fungal and bacterial secretion pathways is established for enzyme production and increasingly applied to other secreted metabolites and bioactive products. These approaches show the shift in secretome research from passive molecular characterization to controlled modification of extracellular signaling outputs. With rigorous characterization and cross-domain comparisons, engineered and primed secretomes can advance regenerative, anticancer, diagnostic, industrial, and agricultural applications.

### 6.2. Inter-Organ Trafficking and In Vivo Secretome Mapping

Inter-organ trafficking refers to the movement of secreted molecules between tissues and organs through the circulation or other biological routes. In vivo secretome mapping aims to identify the cellular or tissue origin of these molecules and, where possible, their distal destinations under physiological or pathological conditions. Circulating proteins can arise from tissue-specific and sometimes unexpected cellular sources, and selected factors may accumulate in tissues distinct from their site of synthesis, suggesting potential roles in inter-organ communication [150,151]. Modern in vivo mapping approaches increasingly use proximity biotinylation with engineered biotin ligases, including TurboID, BirA variants, and endoplasmic reticulum (ER)-targeted systems expressed in a tissue- or cell-type-selective manner [41,152,153,154]. Secreted proteins passing through defined intracellular compartments can thereby acquire biotin labels and subsequently be enriched from plasma, hemolymph, or distal tissues using streptavidin-based capture. Mass spectrometry can then identify and quantify labeled proteins using tandem mass tagging (TMT) or label-free workflows. Cell-type specificity can be achieved using systems such as Cre-dependent expression, Gal4/UAS, tissue-specific promoters, or viral vectors, depending on the experimental model. These workflows are frequently complemented by additional validation and molecular profiling strategies, including:

**(i) Transcriptomic Validation:** Single-nucleus or bulk RNA sequencing can support tissue-of-origin assignments and confirm expression patterns.

**(ii) Genetic Validation:** CRISPR-based knock-ins or tagged endogenous alleles can be used to validate protein localization and trafficking.

**(iii) Biofluid Sampling and Experimental Controls:** Large-scale plasma, hemolymph, or other biofluid collection, together with appropriate controls, can help distinguish secretion, leakage, and alternative release mechanisms.

Notable examples illustrating the power of these approaches include:

***Drosophila* Multi-Omic Secretome Map:** A map of 535 circulating proteins was defined using tissue-specific TurboID labeling across 10 major larval tissues, combined with hemolymph proteomics, snRNA-seq, and approximately 40 knock-in strains. Most identified proteins demonstrated specific origins (including unusual sources) or were identified as shed ectodomains. In vivo experiments confirmed examples of synthesis in one tissue (e.g., fat body or muscle) followed by deposition elsewhere (e.g., cuticle or imaginal discs), providing biochemical evidence consistent with inter-organ trafficking and providing a resource for investigating conserved secreted proteins and potential human homologs [155].

**Mammalian Cell-Type-Selective Profiling:** ER- or membrane-targeted TurboID/BirA systems in mice can enable enrichment of cell-type-labeled proteins from plasma. These approaches can validate known signaling relationships, resolve cell-type-associated secretory profiles, identify unexpected cellular sources of circulating proteins, and detect dynamic changes in secretion under different physiological conditions [150,151].

**Organism-wide and stimulus-responsive maps extend** these systems to multiple cell types and functional tissue states (e.g., exercise training models in mice) to identify stimulus-responsive secreted proteins and candidate inter-organ communication networks [153]. Similar configurations apply to disease models and dietary interventions. Protein-focused proximity labeling is especially useful for assigning cell- or tissue-specific origins to circulating proteins. Complementary approaches such as metabolomics offer direct evidence about the origin, circulation, and tissue distribution of secreted proteins under physiological conditions. Ongoing advancements in labeling enzymes, cell-type coverage, biofluid sampling, and multi-omics integration are expected to enhance the resolution of inter-organ communication maps and clarify changes in secretory networks associated with health and disease.

### 6.3. Single-Cell and Spatial Secretomics

#### 6.3.1. High-Throughput Single-Cell Phenotyping Assay Platforms

Antibody-based multiplexed assays enable the assessment of secretory heterogeneity at the single-cell level. Established approaches include enzyme-linked immunospot (ELISPOT) and FluoroSpot assays, which enable sensitive endpoint detection and enumeration of actively secreting cells. Microengraving, nanowell arrays, and single-cell barcode chips extend these approaches by enabling multiplexed and, in some platforms, dynamic measurement of secreted factors [156,157,158,159].

#### 6.3.2. Mass Spectrometry-Based Single-Cell Approaches

Mass spectrometry (MS)-based approaches provide a discovery-oriented alternative to antibody-based platforms by enabling broader profiling of single-cell secretory outputs. Recent workflows combine metabolic labeling of newly synthesized proteins with bioorthogonal click-chemistry enrichment, followed by in situ enzymatic digestion and nanoflow liquid chromatography–tandem mass spectrometry (nanoLC–MS/MS). One single-cell secretome profiling (SCSP) workflow quantified an average of approximately 389 protein groups per individual HeLa cell secretome and identified 752 proteins across the analyzed population [4]. These approaches can therefore extend proteomic coverage beyond the predefined analyte panels of targeted assays.

#### 6.3.3. Integrated Multi-Omics Systems

Integrated platforms increasingly aim to link transcriptional state with functional secretion at the single-cell level. For example, secretion-encoded single-cell sequencing (SEC-seq) uses functionalized nanovial hydrogel particles to capture secreted factors near individual cells. Researchers can detect captured proteins using oligonucleotide-barcoded antibodies and integrate them with flow cytometry, droplet-based barcoding, and single-cell RNA sequencing. In this way, SEC-seq can associate transcriptomic profiles and surface markers with active secretory phenotypes across large numbers of individual cells [160].

#### 6.3.4. Real-Time and Label-Free Spatiotemporal Imaging

Label-free optical platforms can enable continuous monitoring of secretion dynamics while minimizing the need for exogenous molecular labels. Plasmonic microwell arrays coupled to gold nanohole substrates can use extraordinary optical transmission (EOT) to monitor secreted factors from multiple individual cells in parallel [159]. These systems generate spatiotemporal measurements that integrate signal intensity, cell position, and temporal dynamics while simultaneously tracking cellular morphology and motility.

#### 6.3.5. Spatial Secretomics and Microenvironmental Mapping

Extending secretome analysis from isolated cells to spatially organized tissues enables the investigation of secretion gradients, local cellular interactions, and microenvironmental signaling. Three representative approaches include:

**(i) Secretion Gradient Imaging:** Platforms such as the FluoroDOT assay can visualize and quantify local cytokine secretion around individual cells using plasmonic-fluorescent nanolabels. These approaches can reveal cell-to-cell heterogeneity in secretory responses under different stimulation conditions [161].

**(ii) Contact and Proximity Microchips:** Microfluidic and microchip-based platforms can combine secretome measurements, including soluble proteins and EV-associated signals, with information on physical proximity, migration, and cell–cell contact. Such systems can help resolve how spatial organization influences paracrine signaling and the relative contribution of soluble and vesicular communication [162,163].

**(iii) Tissue-Level Multi-Omics Integration:** Integrating secretomics with spatial proteomics, metabolomics, and transcriptomics can place secretory phenotypes within their anatomical context [162]. This enables analysis of local cell–cell interactions, niche-specific signaling networks, metabolic relationships, and microenvironmental remodeling in complex tissues, including tumor and inflamed immune microenvironments. Collectively, these approaches demonstrate substantial secretory heterogeneity among individual cells, including cells with similar morphological or transcriptional characteristics.

### 6.4. AI-Driven Modeling and Multi-Omics Integration

AI-driven multi-omics integration uses machine learning (ML), deep learning (DL), and foundation models to analyze high-dimensional and heterogeneous biological datasets [49]. These approaches can facilitate dimensionality reduction, cross-modal integration, imputation of incomplete data, and the identification of nonlinear relationships across transcriptomic, proteomic, spatial, and other molecular layers. When applied to secretomics, such models may support the inference of cell–cell communication networks, spatial dependencies, and candidate inter-organ signaling relationships, although these predictions require experimental validation.

#### 6.4.1. Core AI Approaches and Frameworks

**Foundation and Generative Models:** Transformer-based foundation models, such as scGPT, are pretrained on large single-cell datasets to generate gene and cell embeddings [49]. These representations can support transfer learning for cell-type annotation, multi-omics integration, perturbation-response prediction, and gene-network inference involving secretome-associated genes.

**Variational Autoencoders (VAEs):** Multimodal VAEs can learn low-dimensional representations of complex datasets while reconstructing molecular data layers, enabling applications such as modality imputation and joint analysis of transcriptomic and spatial proteomic information. SpaOmicsVAE, for example, combines VAE architectures with graph neural networks and attention mechanisms for spatial multi-omics integration [164].

**Graph Neural Networks (GNNs):** GNNs can model biological relationships represented as graphs, including protein–protein interactions, ligand–receptor relationships, and spatial cellular neighborhoods. Platforms such as COSMOS, SMART, and SpatialCOC integrate spatially resolved multi-omics data while incorporating information on tissue architecture [165,166,167].

**Cross-Modal Integration:** Generative models such as AURORA aim to integrate heterogeneous molecular and clinical data across large cohorts [168]. Such frameworks may enable reconstruction or imputation of missing data layers and support the development of multimodal predictive models for complex biological phenotypes.

**Explainable AI (XAI):** Interpretability approaches aim to identify the features and relationships that contribute to model predictions, including candidate regulators and network interactions. Attention-based methods, graph-based interpretation, and other explainability tools may therefore help generate mechanistic hypotheses from high-dimensional secretome and multi-omics datasets [169].

#### 6.4.2. Functional Applications and Analytical Opportunities

By integrating secretome data with single-cell, spatial, and other multi-omics layers, computational approaches can support ligand–receptor analysis, deconvolution of circulating molecular profiles, and inference of cell-type-associated contributions to extracellular signaling. Predictive models may also help identify stimulus- or treatment-associated changes in secretory output and connect local molecular interactions with broader tissue- and organ-level communication networks [169]. Potential applications include biomarker discovery, therapeutic target prioritization, and developing increasingly detailed computational tissue models.

#### 6.4.3. Current Bottlenecks and Emerging Horizons

Despite rapid methodological development, important challenges remain, including limited training data for rare secretome components, the difficulty of moving from correlative predictions toward causal biological inference, limited interpretability of complex deep-learning models, and the need for rigorous experimental validation. Emerging directions include multimodal foundation models trained across multiple omics layers, federated-learning strategies for distributed clinical datasets, patient-specific computational models or digital twins, and iterative experimental–computational workflows for network discovery [49].

### 6.5. Prioritized Research and Translational Roadmap

Progressing cross-kingdom secretomics from descriptive discovery to predictive and translational applications necessitates a structured framework that integrates biological mapping, functional validation, computational analysis, and product development.

#### 6.5.1. Prioritized Research Directions

To advance secretomics toward predictive and actionable models of biological communication, four research priorities can be identified [42,170,171]:

**High-Resolution Atlases:** Expand cell-type- and tissue-resolved secretome maps to capture rare subpopulations, unconventional secretion pathways, ectodomain shedding, and dynamic changes across physiological and pathological states.

**Functional Validation:** Systematically evaluate candidate secreted signals using genetic perturbation, patient-derived organoids, and complementary functional models to distinguish correlative associations from causal biological relationships.

**Data and Computational Infrastructure:** Develop community-supported standards for data generation, annotation, integration, and cross-batch harmonization, together with interoperable reference datasets and open-access analytical resources.

**Disease-Context Mapping:** Characterize secretome remodeling across health, disease, aging, and therapeutic perturbation, with particular attention to dynamic and tissue-specific communication networks.

#### 6.5.2. Phased Translational Roadmap

Translation of secretome- and EV-based products will require progressive development from product definition and analytical standardization to preclinical evaluation, clinical testing, and regulatory assessment. Table 4 presents a conceptual roadmap highlighting major milestones that may guide this progression.

## 7. Conclusions

### 7.1. Synthesis of the Unifying Perspective

The evidence in this article supports a systems-level view of the secretome as a dynamic, multicomponent network mediating intercellular and inter-organ communication. Recent advances in tissue-specific labeling, single-cell and spatial technologies, multi-omics integration, and computational modeling have greatly improved the investigation of secretory origins, molecular composition, spatial distribution, and context-dependent signaling. Together, these methods are moving the field from fragmented catalogs of secreted factors to comprehensive, integrated maps of extracellular communication.

This broader perspective redefines the secretome as more than a passive molecular readout of individual cell types. It highlights its role as a context-dependent communication system operating across tissues and biological scales. Comprehensive secretome mapping could reveal previously unrecognized signaling relationships, improve biomarker discovery, and support the development of more precisely characterized therapeutic and biotechnological applications.

### 7.2. Paradigm Shift: Moving Beyond the MSC Axis

Historically, regenerative and translational secretome research has mainly focused on mesenchymal stem cell (MSC)-derived secretomes and extracellular vesicles. While this research has provided significant mechanistic and preclinical insights, an MSC-centered view does not fully represent the biological diversity of secretory systems across cell types, tissues, organisms, and disease contexts. Donor variability, tissue-source differences, incomplete molecular characterization, and limited cross-study standardization further emphasize the need for broader, more comparative research. Moving from an MSC-centered framework to cell-type-, tissue-, and organism-resolved secretome mapping may enable several key advancements:

**Discovery of Novel Signaling Sources:** Identification of previously undercharacterized cellular origins of circulating and locally acting factors, including rare or non-stromal populations, unconventional secretion pathways, and inter-organ trafficking relationships.

**Data-Driven Product Design:** Integration of compositional, functional, and multi-omics data to inform the characterization and engineering of secretome-based products.

**Precision and Stratified Applications:** Development of context-specific diagnostic and therapeutic strategies based on tissue origin, molecular cargo, biological activity, and patient- or disease-associated secretory profiles.

Ultimately, moving beyond an MSC-centered paradigm toward standardized, cross-kingdom, and systems-level secretome research may create a more comprehensive framework for understanding extracellular communication. Combining rigorous analytical methods, functional validation, cell- and tissue-resolved mapping, and computational approaches will be essential to translate this biological complexity into reproducible biomarkers, rationally engineered secretome-based products, and more predictive models of intercellular and inter-organ communication.

## Figures and Tables

**Table 1:** Comparative overview of major secretomics workflows ^*a*^.

Workflow	Strengths	Limitations	Best Applicability
**Serum-free MS-based secretomics**	High proteomic coverage; relatively simple workflow	May alter cell physiology; residual intracellular contamination	Mammalian cell lines; MSC studies
**BONCAT/click-chemistry enrichment**	Compatible with serum-containing conditions; high specificity for newly synthesized proteins	Requires optimized labeling conditions; potential toxicity of amino acid analogs	Mammalian cell culture, co-culture systems, and selected microbial models
**Affinity/antibody-based platforms**	High sensitivity for known low-abundance factors	Restricted to predefined targets; dependent on reagent availability and species specificity	Targeted cytokine and growth-factor profiling, particularly in human and mouse systems
**Proximity biotinylation in vivo**	Enables cell-type-of-origin resolution in complex tissues	Requires genetic engineering; potential off-target labeling	In vivo mammalian models and selected multicellular systems
**Plant apoplast/culture secretomics**	Captures both classical and unconventional secretory products	High risk of cytoplasmic contamination; limitations of conventional secretion-prediction tools	Plant secretomics and plant–microbe interaction studies
**Single-cell MS secretomics**	Resolves cell-to-cell heterogeneity in secretory profiles	Currently limited in throughput and proteomic depth; technically demanding	Emerging single-cell applications in mammalian systems

^*a*^ Abbreviations: BONCAT, bioorthogonal non-canonical amino acid tagging; MS, mass spectrometry; MSC, mesenchymal stromal/stem cell.

**Table 2:** Cross-kingdom comparison of secretome frameworks ^*a*^.

Feature	Mammalian Cells	Microbial Contexts	Fungal Contexts	Plant Contexts
**Primary routes**	Classical (ER–Golgi) + UPS + EV biogenesis	Dedicated systems (Types I–IX) + OMVs	Classical + UPS + high-capacity CAZyme secretion	Classical + high proportion of UPS + apoplastic release
**Dominant cargo**	Soluble proteins, cytokines, growth factors, EVs	Toxins, effectors, adhesins, enzymes, metabolites	CAZymes, proteases, secondary metabolites, EVs	Cell-wall enzymes, PR proteins ^b^, peptides, metabolites, EVs
**Regulatory cues**	Hypoxia, inflammation, metabolic state, tissue niche	Nutrient availability, host signals, quorum sensing	Nutrient status, host interaction, envelope stress	Pathogen attack, herbivory, nutrient deficit, symbiotic signals
**Spatial destination**	Local microenvironment + circulome *^c^*	Extracellular milieu, biofilm matrix, host cells	Extracellular space, host tissues	Apoplast + rhizosphere (root exudates)
**Notable adaptations**	Immune modulation and inter-organ communication	Dedicated secretion systems for directional effector delivery	High-capacity secretion of biomass-degrading enzymes	Rigid cell wall constraints; High prevalence of leaderless secreted proteins
**Translational use**	Cell-free therapeutics, liquid biopsy, anticancer	Industrial enzymes, biocontrol, virulence targets	Industrial enzyme cocktails, bioremediation	Bio-stimulants, crop protection, rhizosphere engineering

^*a*^ Abbreviations: CAZyme, carbohydrate-active enzyme; ER, endoplasmic reticulum; EV, extracellular vesicle; OMV, outer-membrane vesicle; UPS, unconventional protein secretion. ^b^ PR proteins refer to pathogenesis-related proteins dynamically upregulated during anti-microbial defenses. *^c^* The circulome represents the systemic, organism-level accumulation of cell-secreted factors detectable in biofluids.

**Table 3:** Quality attributes for secretome products *^a^.*

CQA Category	Recommended Metrics and Assays	Preferred Methods
**Identity & composition**	Proteomic signature, EV surface markers, cargo profile	Quantitative MS, NTA, flow cytometry, multi-omics
**Purity**	Residual host–cell protein or DNA, endotoxin, serum proteins	ELISA, qPCR, LAL assay
**Safety** *^b^*	Adventitious agents, tumorigenicity risk	Sterility testing, mycoplasma testing, in vivo toxicology
**Potency** *^c^*	Mechanism-of-action-linked functional assays	Orthogonal multi-tiered bioassays
**Stability**	Activity retention after lyophilization or storage	Functional potency and cargo integrity over time

*^a^* Abbreviations: CQA, critical quality attribute; ELISA, enzyme-linked immunosorbent assay; EV, extracellular vesicle; LAL, limulus amebocyte lysate; MS, mass spectrometry; NTA, nanoparticle tracking analysis; qPCR, quantitative polymerase chain reaction. *^b^* Safety testing may include assessment of adventitious agents, such as viruses and mycoplasma, according to product type and intended use. *^c^* Orthogonal multi-tiered bioassays for potency include real-time cell proliferation, macrophage polarization, and endothelial tube formation kinetics.

**Table 4:** Conceptual translational roadmap for secretome-based biologics *^a^.*

Phase	Timeline	Strategic Focus and Key Milestones
**Near-Term**	1–3 Years	**Product definition and standardization** Define cell sources, culture parameters, priming/preconditioning conditions, and production media. Develop and validate quantitative, MoA-linked potency assays *^b^*. Conduct multicenter benchmarking of candidate secretome preparations. Develop scalable manufacturing processes and establish appropriate quality-control criteria for clinical-grade production.
**Mid-Term**	3–5 Years	**Preclinical safety and early clinical evaluation** Conduct product-appropriate preclinical safety, biodistribution, and efficacy studies. Advance sufficiently characterized candidates toward early-phase clinical evaluation where appropriate. Validate secretome-derived biomarker panels in independent clinical cohorts. Develop and evaluate multi-omics models for biomarker interpretation and clinical decision support.
**Longer-Term**	5–10+ Years	**Clinical validation and precision applications** Evaluate selected secretome- and EV-based products in later-stage clinical studies, where supported by earlier evidence. Pursue regulatory authorization for products demonstrating adequate quality, safety, and efficacy. Develop precision applications incorporating patient stratification, targeted delivery, and engineered secretome or EV formulations. Integrate validated AI-based and computational modeling approaches into translational workflows. Assess implementation, scalability, and cost-effectiveness across relevant clinical applications.

*^a^* **Abbreviations:** AI, artificial intelligence; EV, extracellular vesicle; MoA, mechanism of action. *^b^* MoA-linked potency assays are intended to complement compositional measurements with functional biological readouts relevant to the proposed mechanism of action.

## Data Availability

No new data were created or analyzed in this study. Data sharing is not applicable to this article. Declaration of AI and AI-Assisted Technologies in the Writing Process: During the preparation of this manuscript, we used AI-assisted tools for technical formatting tasks, such as numbering the reference list and standardizing table styles to meet journal requirements. We also used AI-assisted graphic generation and digital design tools to prepare, refine, assemble, and edit conceptual diagrams in Figure 1, Figure 2, Figure 3 and Figure 4. AI technologies were not used to generate primary scientific data, select literature sources, or formulate the scientific conclusions of this review. The authors reviewed and verified all automated outputs and assume full responsibility for the integrity of the published content.
